# Supplementary material for: The locus coeruleus directs sensory-motor reflex amplitude across environmental contexts
Source: Curr Biol. 2023 Nov 6;33(21):4679–4688.e3. doi: 10.1016/j.cub.2023.08.085 (PMC10957397; doi:10.1016/j.cub.2023.08.085)
Supplement: Document S1. Figures S1–S4 [file mmc1.pdf]

Current Biology, Volume *33*

Supplemental Information

The locus coeruleus directs sensory-motor reflex amplitude across environmental contexts

Emily C. Witts, Miranda A. Mathews, and Andrew J. Murray

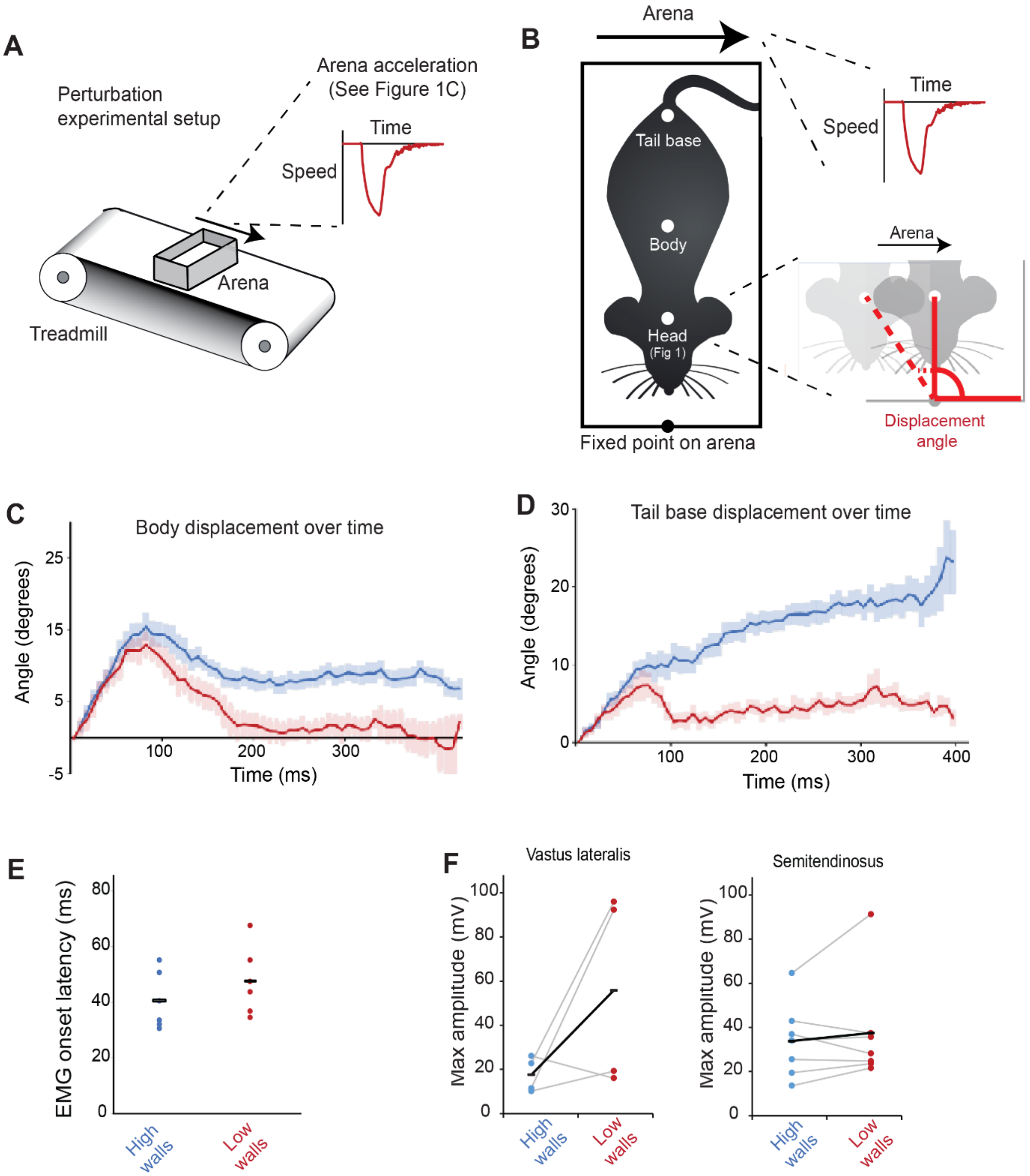

Figure S1: Behavioural setup to assess environmental effects on postural perturbations. Related to Figure 1. (A) Schematic showing experimental setup and perturbation acceleration. (B) Schematic showing placement of tracking dots and measurement of displacement angle. (C) Body displacement angle following perturbation. (D) Tail base displacement following perturbation. (E) Onset latency of muscle response following perturbation in high and low wall conditions. (F) Peak EMG amplitude of VL and ST muscles during perturbation in high and low walled conditions. Each point is the mean of trials from individual experimental animals with the overall mean represented by black lines.

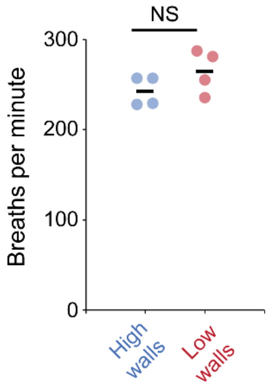

Figure S2: Respiratory rate of mice following lateral perturbations. Related to Figure 1B. Respiratory rate of animals after lateral perturbations.

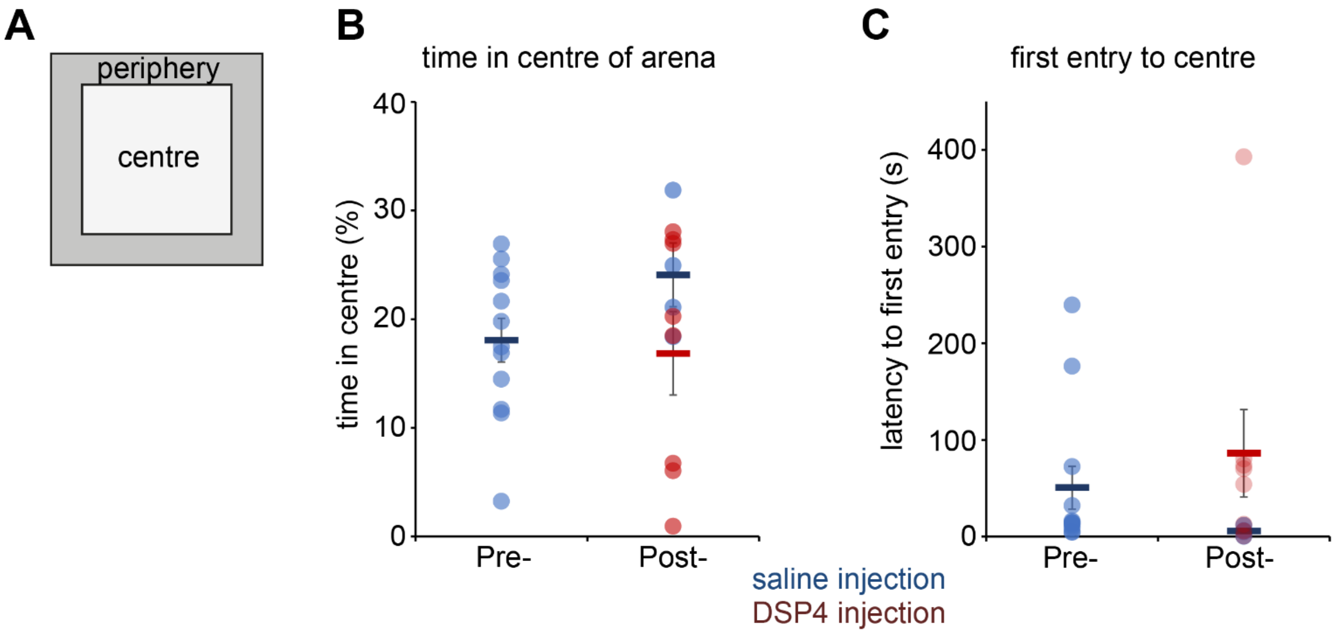

Figure S3: Open field locomotion in DSP4 and control animals. Related to Figure 3. (A) Schematic of open field arena. (B) Percentage of time spent in centre of arena pre- and post- DSP4 or saline injection. (C) Latency of first entry to centre of arena pre- and post- DSP4 or saline injection.

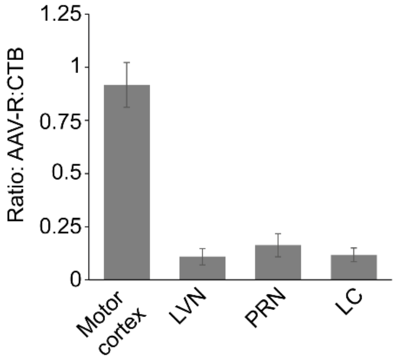

Figure S4: Tropism of AAV2-Retro for distinct populations of spinal projection neurons. Related to STAR methods. AAV-Retro and cholera toxin beta subunit (CTB) were injected into the lumbar spinal cord of adult mice. In motor cortex similar numbers of neurons were observed to contain both CTB and be transduced with AAV-Retro. However, in other regions, including the locus coeruleus, far fewer AAV-retro transduced neurons were observed indicating a lack of tropism for these neurons.
