# Supplementary material for: The locus coeruleus directs sensory-motor reflex amplitude across environmental contexts
Source: Curr Biol. 2023 Nov 6;33(21):4679–4688.e3. doi: 10.1016/j.cub.2023.08.085 (PMC10957397; doi:10.1016/j.cub.2023.08.085)
Supplement: Document S2. Article plus supplemental information [file mmc2.pdf]

Current Biology

Report

The locus coeruleus directs sensory-motor reflex amplitude across environmental contexts

Highlights

- The lateral vestibular nucleus generates motor corrections following perturbation
- This reflex can be altered by manipulating the surrounding environment
- Response adaptation is influenced by noradrenergic signals from the locus coeruleus

Authors

Emily C. Witts, Miranda A. Mathews, Andrew J. Murray

Correspondence

e.reader-harris@ucl.ac.uk (E.C.W.),
a.murray@ucl.ac.uk (A.J.M.)

In brief

Witts et al. show that motor corrections generated by the lateral vestibular nucleus can be altered by manipulating the surrounding environment, and this environmental influence requires noradrenergic signaling from the locus coeruleus.

Witts et al., 2023, Current Biology *33*, 4679–4688
November 6, 2023 © 2023 The Authors. Published by Elsevier Inc.
https://doi.org/10.1016/j.cub.2023.08.085

CellPress

Current Biology

CellPress
OPEN ACCESS

Report

The locus coeruleus directs sensory-motor reflex amplitude across environmental contexts

Emily C. Witts,[1,*] Miranda A. Mathews,[1] and Andrew J. Murray[1,2,3,*]
[1]Sainsbury Wellcome Centre for Neural Circuits and Behaviour, University College London, W1T 4JG London, UK
[2]Present address: Sania Therapeutics, Waterfront Building, Manbre Wharf, London, W6 9RH, UK
[3]Lead contact
*Correspondence: e.reader-harris@ucl.ac.uk (E.C.W.), a.murray@ucl.ac.uk (A.J.M.)
https://doi.org/10.1016/j.cub.2023.08.085

SUMMARY

Purposeful movement across unpredictable environments requires quick, accurate, and contextually appropriate motor corrections in response to disruptions in balance and posture.[1–3] These responses must respect both the current position and limitations of the body, as well as the surrounding environment,[4–6] and involve a combination of segmental reflexes in the spinal cord, vestibulospinal and reticulospinal pathways in the brainstem, and forebrain structures such as the motor cortex.[7–10] These motor plans can be heavily influenced by the animal's surrounding environment, even when that environment has no mechanical influence on the perturbation itself. This environmental influence has been considered as cortical in nature, priming motor responses to a perturbation.[8,11] Similarly, postural responses can be influenced by environments that alter threat levels in humans.[12–18] Such studies are generally in agreement with work done in the mouse showing that optogenetic stimulation of the lateral vestibular nucleus (LVN) only results in motor responses when the animal is on a balance beam at height and not when walking on the stable surface of a treadmill.[10] In general, this ability to flexibly modify postural responses across terrains and environmental conditions is a critically important component of the balance system.[19,20] Here we show that LVN-generated motor corrections can be altered by manipulating the surrounding environment. Furthermore, environmental influence on corrections requires noradrenergic signaling from the locus coeruleus, suggesting a potential link between forebrain structures that convey sensory information about the environment and brainstem circuits that generate motor corrections.

RESULTS

Environment influences the motor response to a perturbation

Vestibulospinal reflexes act to maintain balance and upright posture. The LVN, and its spinal projection from lateral vestibulospinal tract (LVST) neurons, is a key brainstem structure required for the generation of a reflexive motor correction following a postural perturbation.[10] To ascertain whether these postural reflexes could be influenced by environmental context, we developed a behavioral paradigm that could alter perceived threat levels as mice underwent a perturbation.

Rodents are known to avoid open spaces due to the possibility of predation and show a preference for enclosed spaces, particularly in novel environments.[21] This is exemplified in rodent behavioral tests of anxiety, such as the elevated plus maze or thigmotaxis in an open field arena.[22–24] Using this information as a starting point, we designed a behavioral paradigm where mice would undergo a lateral postural perturbation, forcing them to make a corrective motor response to maintain an upright posture. Mice received this perturbation in an enclosure measuring 10 cm by 6 cm in the XY plane. In order to test whether the external environment could influence the postural response, we varied the height of the enclosure in the Z plane. A "low-wall" condition, where the height of the enclosure was slightly above head height of the animal, was compared to a "high-wall" condition, which was double the height (3.5 vs. 7 cm; Figure 1A). We hypothesized that mice would feel more exposed or threatened in the low-wall condition. Consistent with this, we observed the respiratory rate of mice (a proxy for anxiety)[25] to be elevated in the low-wall condition (220.5 ± 7.5 breaths per minute) compared to the high-wall condition (180.1 ± 14.6 breaths per minute; $n = 4$ mice; $p = 0.049$; Figure 1B). A perturbation was then introduced by rapid lateral movement of the entire arena. We measured the movement of each arena to ensure animals received identical perturbations in the two environments. The displacement of each arena was 115 mm in the lateral plane with a peak acceleration between 0.53 and 0.54 ms, reaching 50 ms from movement onset. The entire movement lasted 140 ms (Figures 1C, S1A, and S1B). Following perturbation, there was no longer a significant difference in breathing rate between animals in the high- and low-wall conditions (Figure S2).

We closely monitored the postural correction initiated by the mice following the perturbation. We measured the displacement of a fixed point on the head over time, starting at perturbation onset (time 0; Figures 1D, 1E, and S1B) relative to a point within the arena. Animals in the low-wall environment were displaced

This is an open access article under the CC BY license (http://creativecommons.org/licenses/by/4.0/).

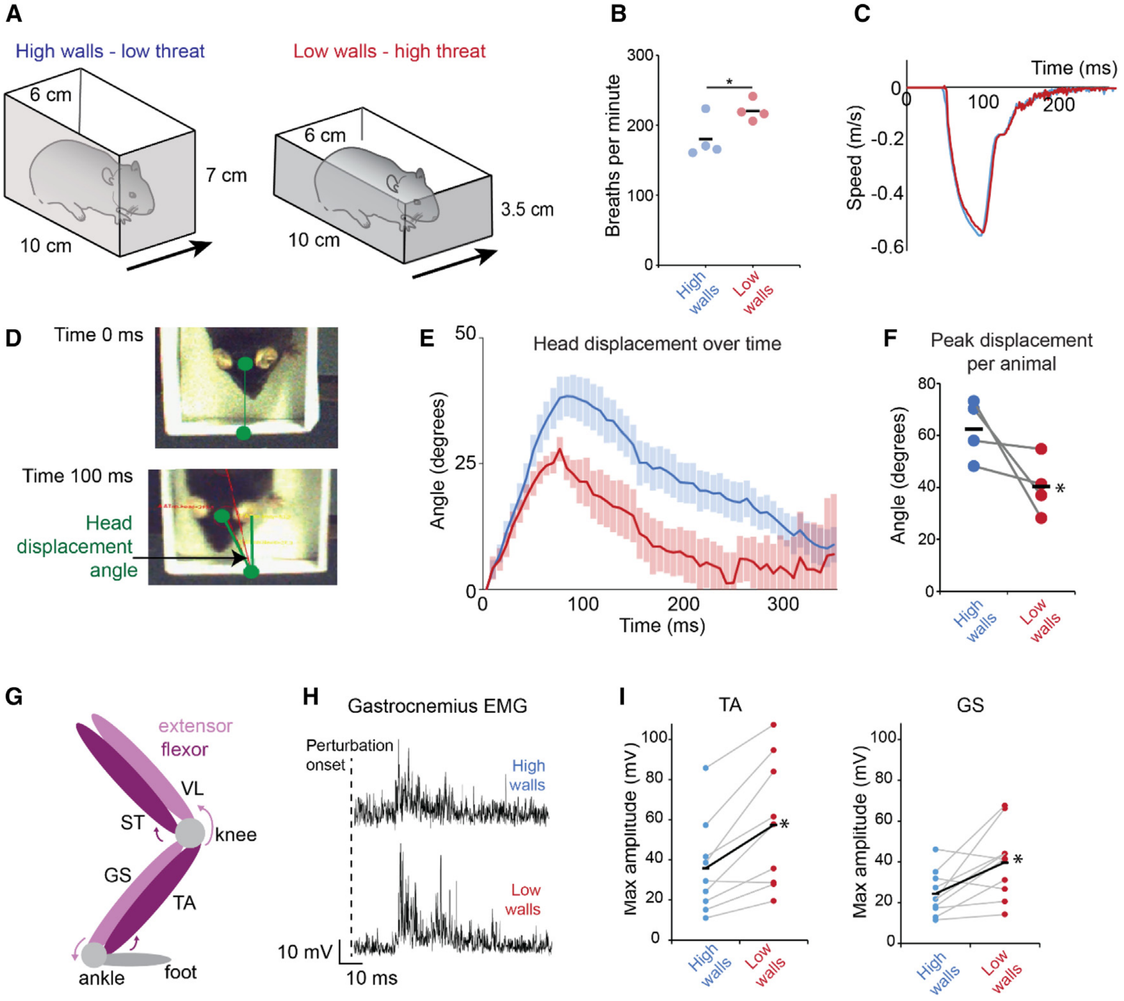

Figure 1. Environmental context alters response to unexpected perturbation

(A) Schematic showing the behavioral apparatus to vary environmental context.
(B) Breaths per minute in experimental animals in the high- and low-wall conditions.
(C) Traces of the arena displacement (perturbation) in the high- and low-wall conditions.
(D) Example images of head position relative to fixed point in the arena prior to perturbation onset (time 0) and 100 ms after perturbation onset.
(E) Change in head displacement angle over time from perturbation onset (time 0) in high- and low-wall conditions.
(F) Peak head displacement angle after perturbation in high- and low-wall conditions.
(G) Diagram showing location of hindlimb muscles implanted with electrodes (see also Figure S1).
(H) Example rectified EMG traces from the GS muscle in high- and low-wall conditions after perturbation onset.
(I) Peak EMG amplitude of TA and GS muscles during perturbation in high- and low-wall conditions. Each point is the mean of trials from individual experimental animals with the overall mean represented by black lines. *$p < 0.05$.
See also Figures S1 and S2.

significantly less than those in the high-wall condition (Figure 1E), consistent with a stronger and more effective postural correction. The peak displacement of the head (defined as the maximum angle reached by the head compared to a fixed point on the arena) for the high-wall condition was 62.4° ± 5.8° compared with only 40.3° ± 5.5° for the low-wall condition ($p = 0.032$; Figure 1F). Similarly, fixed points on the body and base of the tail showed decreased stability in the high-wall condition over the 400 ms recording period (Figures S1C and S1D).

To examine limb muscle activity underlying the difference in body movement, we performed electromyography (EMG) recordings from the extensor muscles gastrocnemius (GS) and vastus lateralis (VL) and the flexor muscles tibialis anterior (TA) and semitendinosus (ST) in the hindlimb (Figure 1G). Consistent with a stronger postural correction, the peak EMG amplitude in ankle muscles GS and TA were larger in the low-wall condition (39.7 ± 5.6 mV; n = 10 mice and 57.4 ± 10.7 mV; n = 9 mice) compared to the high-wall condition (24.4 ± 3.4 mV; n = 10 mice; p = 0.0023 and 35.9 ± 7.9 mV; n = 9 mice; p = 0.016; Figures 1H, 1I, and S1F). The latency of onset of the EMG response (measured as time from perturbation onset to the peak response; Figure S1E) was not

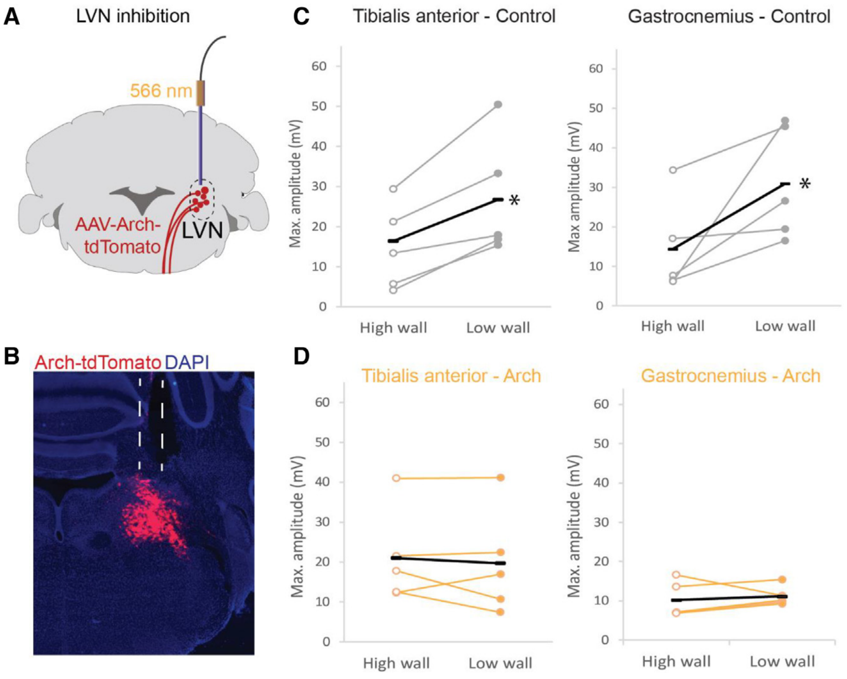

Figure 2. Inhibition of the LVN reduces response to perturbation

(A) Schematic showing experimental strategy to transiently inhibit neurons in the LVN.

(B) Histological image showing virus injection in the LVN and placement of fiber optic cannula.

(C) Mean peak EMG amplitude after perturbation without light (control).

(D) Mean peak EMG amplitude after perturbation with light on and inhibition of LVN neurons. In (B) and (D), each point is the mean of trials from individual experimental animals with the overall mean represented by black lines. LVN, lateral vestibular nucleus. *$p < 0.05$.

different between conditions, indicating that only the amplitude and not the timing of the EMG response was influenced by the environment.

Silencing of the LVN prevents postural corrective responses

Vestibulospinal pathways convey proprioceptive and vestibular sensory information to spinal motor circuits,[26] and these pathways are required for postural corrections in mice.[10] However, local reflexes at the level of the spinal cord also contribute to postural control.[2] To test whether the LVN was required for the generation of postural reflexes in our behavioral assay, we inhibited the activity of the LVN during lateral perturbations (Figures 2A–2D). We stereotaxically injected an adeno-associated virus (AAV) expressing the outward proton pump archaerhodopsin (ArchT)[27] into the LVN and implanted a fiber optic probe to deliver yellow light to neurons in this area (Figures 2A and 2B), selectively and reversibly inhibiting the LVN during postural perturbations.

When neurons in the LVN were inhibited using ArchT, the response to a lateral perturbation was substantially altered. In TA, without light, there was an elevated response in low- (26.8 ± 6.7 mV; n = 5 mice) compared to high-wall (14.8 ± 4.7 mV; n = 5 mice; Figure 2C) conditions, as observed above. However, in the same animals, the presence of light to silence cells in the LVN caused a reduced response to perturbation in both the high- (21.4 ± 6.3 mV; n = 5 mice) and low-wall (19.7 ± 5.9 mV; n = 5 mice) conditions (Figure 2D). Similarly, in GS, in the absence of light, amplitude of EMG signals in response to perturbation was larger in the low-wall condition (30.9 ± 6.4 mV; n = 5 mice) compared to the high-wall condition (14.3 ± 5.4 mV; n = 5 mice; Figure 2C). When the light was on, however, and neurons in the LVN were inhibited, there was a reduced response in both the low- (11.1 ± 1.1 mV; n = 5 mice) and high-wall (10.2 ± 2.0 mV; n = 5 mice) conditions (Figure 2D).

Noradrenergic signaling contributes to normal locomotor activity

The results above, along with previous work,[10,28] indicate that vestibulospinal neurons are required for the muscular response to counteract a perturbation. Our behavioral paradigm indicates that this response can be varied depending on the environmental context, but how does information regarding the surrounding environment feed into postural circuits?

One possibility is the involvement of noradrenergic signaling. Previous studies looking at vestibulospinal reflexes in decerebrate cats have indicated that noradrenaline can influence vestibulospinal reflex gain.[28–30] The locus coeruleus (LC) has been suggested to be the source of this noradrenergic input to the LVN and is also known to have further roles in overall posture and arousal.[31] Given that the LC is implicated in attention and vigilance,[32] we hypothesized that this region could provide the necessary link between environmental context and postural control pathways.

Studies in reduced preparations have demonstrated that noradrenergic activity is involved in the initiation and modulation of locomotion[33] and can directly influence motor neuron activity.[34] Therefore, prior to assessing postural control, we first examined whether the blockade of noradrenergic signaling resulted in any gross motor changes. To achieve this, we selectively disrupted noradrenergic signaling with the compound N-(2-chloroethyl)-N-ethyl-2-bromobenzylamine (DSP-4),[35] which causes toxin buildup in cells, leading to the destruction of noradrenergic terminals as well as irreversibly blocking noradrenergic transporters.[36,37] To test for gross motor problems, we first examined mice in an open field arena. Wild-type mice (n = 12) first underwent habituation sessions in the arena, followed by trials where both path length and velocity were measured. Animals then received either an intraperitoneal injection of 5 mg/mL of DSP-4 (final concentration of 50 mg/kg; n = 8) or vehicle (saline; n = 4) as a control 7 days prior to further behavioral testing (Figure 3A).

Consistent with previous reports,[38] we observed a ~25% reduction in path length when comparing the same group of animals pre- and post-toxin injection (p = 0.031) and a ~32%

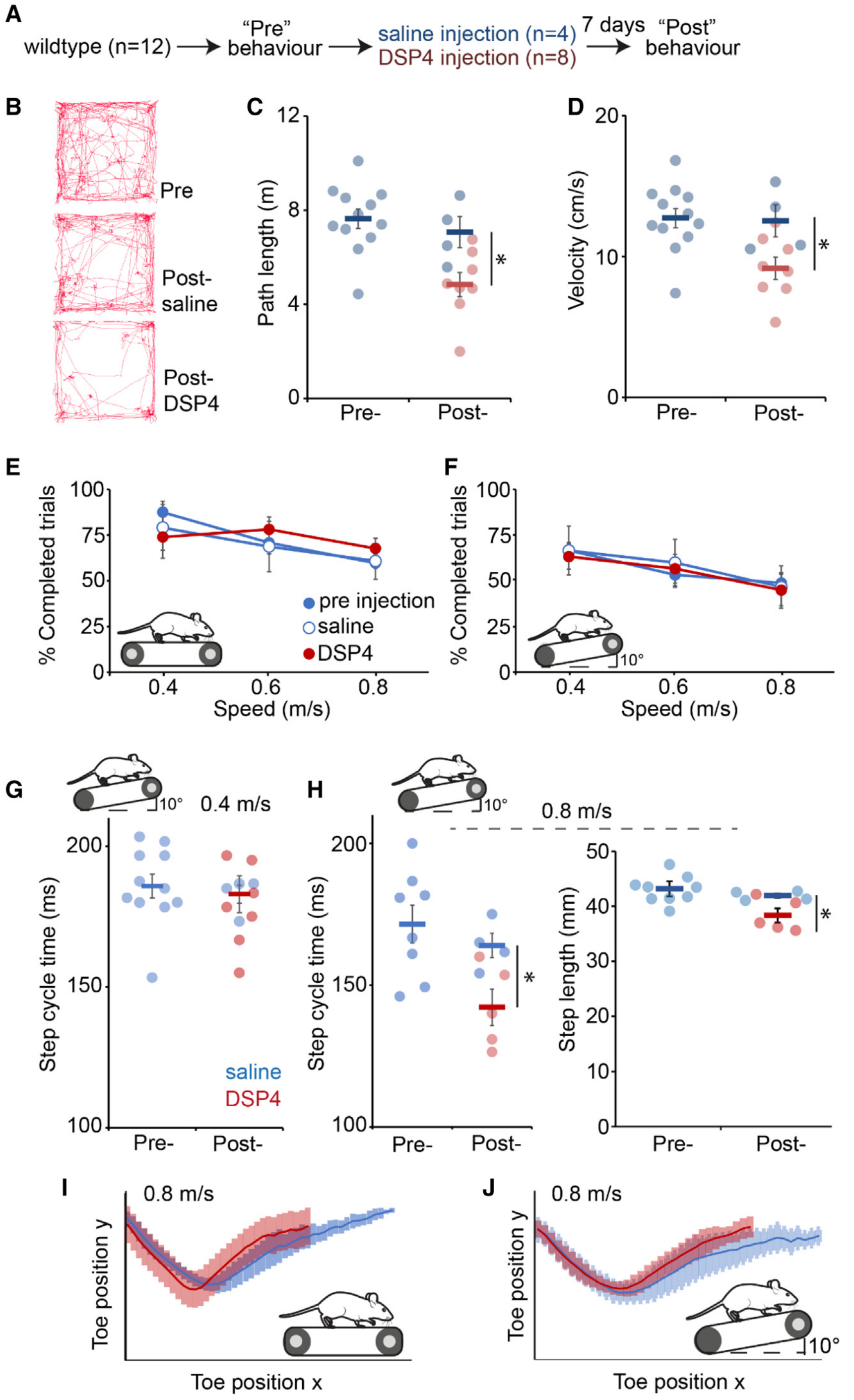

Figure 3. Disruption of the LC specifically affects strenuous locomotion

(A) Experimental plan for disruption of noradrenergic signaling via the injection of the selective noradrenergic neurotoxin DSP-4.
(B) Representative path lengths in 10 min open field.
(C) Overall path lengths in open field.
(D) Locomotor velocity in open field.
(E) Ability of control and DSP-4-injected animals to maintain consistent speed on a horizontal treadmill at 0.4, 0.6, and 0.8 m/s.
(F) Ability of wild-type and DSP-4-injected animals to maintain consistent speed on an inclined treadmill at 0.4, 0.6, and 0.8 m/s.
(G) Step cycle time on incline treadmill at 0.4 m/s.
(H) Step cycle time (left) and step length (right) on incline treadmill at 0.8 m/s.
(I) Toe position in xy coordinates over the step cycle at 0.8 m/s on horizontal treadmill.
(J) Toe position in xy coordinates over the step cycle at 0.8 m/s on inclined treadmill. *$p < 0.05$.
See also Figure S3. Error bars are ± SEM.

reduction in path length when comparing DSP-4 and saline injected animals ($p = 0.027$; Figures 3B and 3C). A reduction of path length over the 5 min open field recording could be the result of DSP-4-treated animals having overall slower locomotion or spending more time inactive and not moving. The latter could be an indication of anxiety due to disrupted noradrenergic signalling.[39] To address this, we examined the velocity of animals during periods of movement in the open field and found that DSP-4-treated animals had significantly slower movements when compared within groups pre- and post-toxin injection ($p = 0.032$) or between groups after DSP-4 or saline injection ($p = 0.034$; Figure 3D). In addition, we did not observe any alterations in time spent in the center of the open field arena, which is an indicator of anxiety levels (Figure S3).

Next, we examined treadmill running in animals treated with DSP-4. First, we examined the ability of DSP-4-treated and control animals to maintain a constant speed on a horizontal or inclined treadmill. All groups were equally able to maintain walking on a horizontal (Figure 3E) or inclined (Figure 3F) treadmill for 3 s at 0.4, 0.6, and 0.8 m/s. This suggests that overall locomotor ability is not dependent on noradrenergic signaling.

In addition to their ability to maintain a constant speed, we also examined aspects of locomotor kinematics across the various conditions. On an inclined treadmill at 0.4 m/s, the step cycle duration of control and DSP-4-treated animals was not different between groups (pre-injection = 185.7 ± 3.1 ms; DSP-4 = 183.2 ± 4.2 ms; n = 12 mice; $p = 0.6$; Figure 3G). However, at the faster speed of 0.8 m/s, we noted that DSP-4-treated animals had an approximately 15%–20% shorter step cycle time when compared to control groups (Figure 3H) (pre-injection = 171.5 ± 6.7 ms; saline injection = 164.0 ± 4.3 ms; DSP-4 injected = 142.2 ± 6.4 ms; main effect of group, $F = 5.3$, $p = 0.019$). Similarly, DSP-4-injected animals took significantly shorter steps than saline-injected controls (saline = 41.9 ± 0.4 mm; DSP-4 = 38.3 ± 1.3; $p = 0.049$; Figure 3H). Kinematic analysis of both horizontal and incline treadmill walking at

0.8 m/s also showed shorter steps under both conditions (Figures 3I and 3J).

Overall, these locomotor results show that noradrenergic signaling is important only for high intensity locomotion, namely incline running at high speed. This could indicate a role for the LC in maintaining a high gain of locomotor activity.

The LC sets the level of motor response to a perturbation

We next examined whether noradrenergic signaling played a role in the generation of postural reflexes. We first looked at noradrenergic input to the LVN by examining tyrosine hydroxylase (TH), which is present in noradrenergic and dopaminergic neurons, immunostaining in the LVN. We observed extensive TH labeling in axons throughout the LVN in close apposition to LVST neurons labeled via cholera toxin beta (CTb) injected into the lumbar spinal cord (Figure 4A), suggesting noradrenergic input to the LVN, as has been shown previously in rats.[40]

Next, we examined whether pharmacological disruption of noradrenergic neurons would affect the EMG response to a perturbation in different environmental conditions. Again, we systemically administered DSP-4 to disrupt noradrenergic neurotransmission (Figure 4B). In the TA muscle, before administration of DSP-4, EMG peak amplitudes were greater in low- (34.3 ± 13.9 mV) than high-wall (16.0 ± 4.2 mV; $p = 0.044$) conditions. After systemic administration of DSP-4, similar peak EMG amplitudes were recorded in mouse hindlimb muscles in the high- (32.2 ± 6.4 mV) and low-wall (35.3 ± 14.6 mV) conditions following perturbation ($p = 0.73$; Figures 4B–4D). These results are consistent with a potential role for noradrenergic signaling in setting the gain of a postural response.

Although DSP-4 is a potent neurotoxin for noradrenergic neurons of the LC, resulting in ~90% neuron ablation, it has been reported to ablate up to 50% of neurons in other noradrenergic nuclei, including those that innervate the spinal cord.[41] To ascertain whether the alterations in postural corrections are indeed coordinated by the LC, and not by other noradrenergic nuclei, we used a viral strategy for selective blockade of neurotransmission from only those neurons. We took advantage of a previously reported noradrenergic selective promoter (PRSx8, a synthetic promotor containing Phox2B binding motifs),[42] which we cloned and packaged into an AAV. We first assessed the specificity of the PRSx8 promoter in an AAV by placing it upstream of a GFP reporter, which was packaged into an AAV-DJ capsid (Figure 4E). Stereotaxic injection of this vector into the LC of wild-type mice resulted in 61.6%+/−8.9% of LC neurons expressing GFP (Figures 4F–4I). In contrast, 20.3% ± 8.6% of GFP-positive neurons did not express TH (though this could represent neurons expressing low levels of TH) (Figure 4J).

We next packaged an AAV expressing cre recombinase under control of the PRSx8 promoter and co-injected this along with an AAV containing a cre-conditional tetanus toxin light chain fused to GFP or GFP alone (Figure 4K).[43] The tetanus toxin light chain prevents synaptic transmission through disruption of vesicle docking. In these experimental conditions, synaptic transmission is selectively blocked from noradrenergic neurons in the LC only (Figures 4K–4M). Control animals expressing GFP in the LC showed an increased EMG amplitude in response to perturbation in low- vs. high-wall conditions in the TA muscle (high mean max. amplitude 38.8 ± 4.4 mV; low mean max. amplitude 66.5 ± 9.7; $n = 5$ mice; $p = 0.048$; Figure 4N). However, the blockade of synaptic transmission resulted in highly similar EMG responses in high- and low-wall conditions (high mean max. amplitude 53.0 ± 4.7 mV; low mean max. amplitude 53.6 ± 5.3; $n = 5$ mice; $p = 0.89$; Figure 4O). Therefore, in agreement with our data using DSP-4, the selective blockade of synaptic transmission from the LC results in postural responses no longer being tuned to the environment. Overall, these results support a model whereby fast postural corrections are initiated by the LVN, and the LC sets the level of this response according to the environmental context.

DISCUSSION

In order to maintain upright posture, the nervous system must quickly respond to mechanical perturbations that impact the body and produce a counteracting motor output. The motor response can change according to the environmental context and can be heavily influenced by perceived threat. Here we have shown that the LC can alter the gain of vestibulospinal reflexes that maintain balance following a postural perturbation. This noradrenergic influence on motor circuits provides a potential link between forebrain regions that interpret environmental context and the sensory-motor reflex pathways of the brainstem and spinal cord.

In response to an unexpected postural challenge or perturbation, animals and humans must generate a motor response that counteracts the displacement. Rather than this being a simple motor reflex, it is well known that these postural corrections are adapted to suit a range of environmental and spatial contexts.[8,44,45] For example, the gain of postural reflexes can be altered in humans when they are standing on the edge of a high platform.[16] Evolutionarily, this mechanism could indicate the nervous system focusing more on postural reflexes under circumstances where the cost of falling is extremely high or maintaining a stable body position under threatening conditions.[14,46] This can be either by a priming of body position by altering muscle tension or by a heightened awareness stored neuronally, which feeds into motor responses to external stimuli. To probe the circuit mechanisms that link the environment to motor reflexes, we developed a mouse behavioral assay that allows for adaptation of postural reflexes in different environmental contexts. When in unfamiliar environments, mice tend to prefer enclosed spaces, as observed by thigmotaxis in open field arenas or the preference for enclosed areas on a plus maze.[24] We reasoned that mice in more exposed environments would experience higher threat and stress levels than when in a more enclosed space, and that this threat could mimic that experienced by humans when standing at height.[16] By altering the height of the surrounding walls on a moving platform, we could alter the motor correction and muscle response. Specifically, when in a more open environment, the amplitude of the muscle response to the perturbation (as measured via EMG) was increased, resulting in a greater postural correction and reduced body sway even when the perturbation was of equal magnitude across the two environments. Using a combination of disruption of noradrenaline transmission and direct targeting of the LC, we

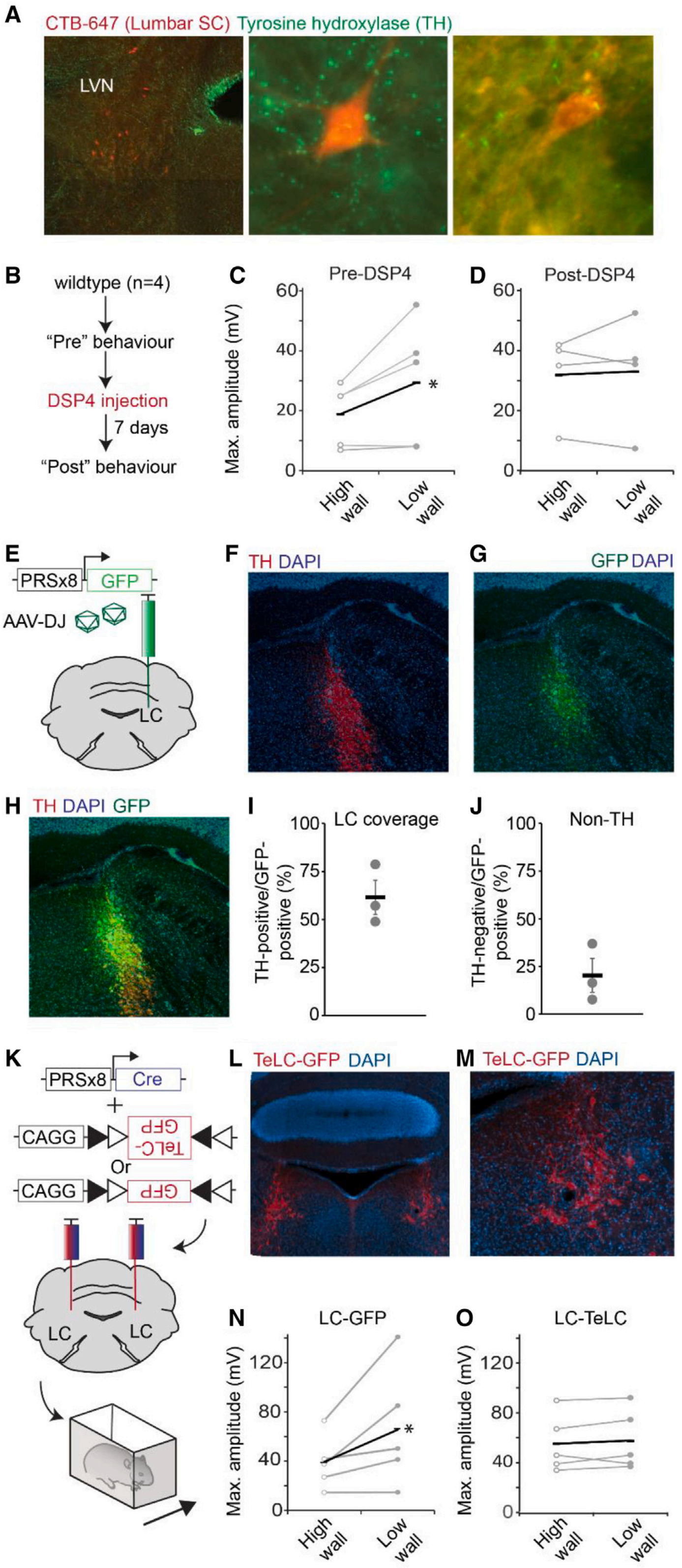

Figure 4. The LC is involved in processing information regarding environmental context

(A) LVST neurons labeled via CTB-647 injection into the lumbar spinal cord and sections stained with anti-TH. Higher magnification images (middle and right) show apposition of TH-positive enlargements in the vicinity of LVST neurons.
(B) Experimental procedure to test effect of noradrenergic neurotoxin DSP-4 on responses to lateral perturbations.
(C) Comparison of peak EMG amplitudes in TA after perturbation in control animals in high- and low-wall conditions.
(D) Comparison of peak EMG amplitudes in TA muscle after perturbation in animals following DSP-4 injection in high- and low-wall conditions.
(E) Experimental procedure for selective labeling of noradrenergic neurons in the LC using the PRSx8 promoter.
(F) TH immunostaining at site of injection of AAV described in (E).
(G and H) GFP labeling and (H) merge of same site shown in (F).
(I) Proportion of TH-positive neurons in the LC that express GFP following AAV injection.
(J) Proportion of TH-negative neurons expressing GFP.
(K) Experimental strategy for blocking synaptic transmission from neurons in the LC.
(L) Bilateral targeting of TeLC-GFP to the LC.
(M) Higher magnification of image in (L).
(N) Peak EMG responses of TA muscle in response to perturbations in high- or low-wall conditions in animals expressing GFP in the LC.
(O) Peak EMG responses of TA muscle in response to perturbations in high- or low-wall conditions in animals expressing GFP-TeLC in the LC.
Error bars are ± SEM

showed that this effect is mediated by noradrenergic signaling from the LC.

Noradrenaline is known to be released in response to stress or in situations of heightened arousal,[47] and circuits linking the LC and the forebrain have indicated that noradrenaline has a role in attention and vigilance.[32] Our prevailing hypothesis is that the increased breathing rate shown in the low-wall condition demonstrates raised anxiety levels, which are encoded by noradrenergic signaling from the LC. However, the breathing rate and/or anxiety levels of the mice may be unrelated to the change in perturbation response, and the effect may be a result of changed visual stimuli or other environmental factors, and further study will be required to test this hypothesis directly. Indeed, stress and anxiety signaling is not the only role of noradrenaline in the nervous system. Classical studies in decerebrate animals have indicated that noradrenaline can play a facilitatory role in postural responses[28] and can influence the gain of vestibulospinal reflexes[48]; in addition,cessation of activity in the LC has been observed during loss of muscle tone in cataplexy.[49] Noradrenergic signaling can be facilitatory or inhibitory, depending on the postsynaptic receptor complement. In the cat, the microiontophoretic injection of noradrenaline into the LVN increases resting discharge of the neurons, whereas, conversely, in the adjacent medial vestibular nucleus it decreases resting activity.[50] The LC can also influence spinal circuits directly via coeruleospinal pathways, though this pathway has been predominantly implicated in nociceptive processing.[51] In addition, the LC itself is known to receive input from a diverse array of brain regions, including the prefrontal cortex, hypothalamus and amygdala,[52] which could inform the postural control system of the surrounding environment. These sensory-motor relationships provide a potential circuit whereby higher order information regarding the nature of the surrounding environment is routed through the LC to motor pathways of the LVN.

Disruption of noradrenergic signaling had minor effects on locomotor activity. Noradrenaline is known to increase or sustain locomotor activity in isolated spinal cord preparations[53] and whole organisms,[38,54] which may be related to the changes in motor activity at high speeds and on an incline that we observe here (Figure 3). In agreement with previous studies, we observed a reduced open field path length after injection of DSP-4.[55] Additionally, in a task that required increased locomotor effort, we did observe that mice with disrupted noradrenergic signaling had altered stepping (shorter and faster steps) when compared to control animals. Potentially, this could indicate noradrenergic signaling also feeds into locomotor circuits under circumstances that require an enhanced motor output.

There are several environmental factors that may influence the results presented here. There may be some influence of a change in visual input, in particular, the differences in optic flow in high- and low-wall conditions. Though we did not observe alterations in response levels across trials, habituation to perturbation could also influence the muscle responses recorded. For example, if the mouse habituated to the environment over time, leading to altered stress levels at different points in the experiment. It would be interesting to see if the results obtained here are altered by recording in the dark or with an artificially imposed visual input. In addition, though we controlled for starting posture of the mouse by visualizing body and head position, we were unable to visualize the exact foot placement of the animals. The link between foot placement and postural response is well known to influence postural responses[56–58] and could be studied in our paradigm using a clear treadmill and a ventrally located camera.

Clinically, degeneration of the LC in Parkinson's disease has been linked to problems with balance.[59,60] Some aspects of Parkinsonian postural responses—such as an increased amplitude of the medium latency postural response—are reminiscent of the results obtained in our study.[20,61] Additionally, Parkinsonian patients show a reduction in locomotor step length and velocity.[62] Furthermore, drugs that raise noradrenaline levels are related to increased risk of falls.[63] Interestingly, gait disorders and an increase in falls are particularly clinically challenging because they do not tend to improve with the common therapeutic interventions levodopa and deep brain stimulation.[64,65] However, treatment of a rat model of Parkinson's disease with noradrenaline reuptake inhibitors alone or in combination with alpha2 receptor antagonists can rescue some motor deficits[66] of the disorder. Coupled with our results, this could indicate that disruption of noradrenergic signaling in Parkinson's disease could contribute to the balance and motor deficits by altering the activity of vestibulospinal pathways.

At a circuit level, we suggest two potential mechanisms whereby LC activity could influence postural control. First, in the event of an unexpected perturbation, the LC uses information about the environment to fire phasically with the LVN, which then implements the motor correction at the level of the spinal cord. Alternatively, the LC is informed of the change in context prior to any perturbation and alters motor output either via the LVN or directly through spinal motor neurons via the coeruleospinal pathway. This second model would be consistent with the known role of the LC in stress, where sensitivity to hormonal changes means that LC firing tends to increase tonically rather than in response to discrete stimuli,[67–69] and so it may have a similar role in the postural control system. Though we attempted to ascertain the role of coeruleospinal neurons in postural control in our paradigm, technical limitations in viral tropism (see STAR Methods) prevented us from targeting this population directly. In future studies, it will be important to ascertain the exact area of the LC circuit that mediates postural control.

STAR★METHODS

Detailed methods are provided in the online version of this paper and include the following:

- KEY RESOURCES TABLE
- RESOURCE AVAILABILITY
  - Lead contact
  - Materials availability
  - Data and code availability
- EXPERIMENTAL MODEL AND STUDY PARTICIPANT DETAILS
- METHOD DETAILS
  - Surgical procedures
  - Behavioral procedures
  - Recording techniques
  - Viruses

- ○ Retrograde labeling
- ○ Drugs
- ○ Histology
- ● QUANTIFICATION AND STATISTICAL ANALYSIS
  - ○ Data analysis

SUPPLEMENTAL INFORMATION

Supplemental information can be found online at https://doi.org/10.1016/j.cub.2023.08.085.

ACKNOWLEDGMENTS

We thank Dr. Turgay Akay (Dalhousie University), Dr. Niccolo Zampieri (MDC Berlin), Prof. Rob Brownstone (University College London), and members of the Murray lab for comments on the manuscript. This work was supported by Wellcome grant 219627/Z/19/Z and Gatsby Charitable Foundation grant GAT3755 (both Sainsbury Wellcome Centre [SWC] core grants). An image from SciDraw made by Gil Costa (scidraw.io) was used in Figure S1B.

AUTHOR CONTRIBUTIONS

E.C.W. devised the concept, designed the experiments, performed the experiments, analyzed the data, and wrote the manuscript. M.M. performed experiments and analyzed data. A.J.M. devised the concept, designed the experiments, performed experiments, analyzed the data, and wrote the manuscript.

DECLARATION OF INTERESTS

The authors declare no competing interests.

Received: June 7, 2023
Revised: July 3, 2023
Accepted: August 25, 2023
Published: September 22, 2023

REFERENCES

1. Carpenter, M.G., Allum, J.H., and Honegger, F. (1999). Directional sensitivity of stretch reflexes and balance corrections for normal subjects in the roll and pitch planes. Exp. Brain Res. *129*, 93–113. https://doi.org/10.1007/s002210050940.
2. Deliagina, T.G., Beloozerova, I.N., Zelenin, P.V., and Orlovsky, G.N. (2008). Spinal and supraspinal postural networks. Brain Res. Rev. *57*, 212–221. https://doi.org/10.1016/j.brainresrev.2007.06.017.
3. Denny-Brown, D.E. (1929). The Histological Features of Striped Muscle in Relation to its Functional Activity. Proc. Royal Society *104*, 371–411.
4. Balasubramaniam, R., and Wing, A.M. (2002). The dynamics of standing balance. Trends Cogn. Sci. *6*, 531–536. https://doi.org/10.1016/s1364-6613(02)02021-1.
5. Cleworth, T.W., Horslen, B.C., and Carpenter, M.G. (2012). Influence of real and virtual heights on standing balance. Gait Posture *36*, 172–176. https://doi.org/10.1016/j.gaitpost.2012.02.010.
6. Macpherson, J.M., Horak, F.B., Dunbar, D.C., and Dow, R.S. (1989). Stance dependence of automatic postural adjustments in humans. Exp. Brain Res. *78*, 557–566. https://doi.org/10.1007/BF00230243.
7. Deliagina, T.G., Zelenin, P.V., Beloozerova, I.N., and Orlovsky, G.N. (2007). Nervous mechanisms controlling body posture. Physiol. Behav. *92*, 148–154. https://doi.org/10.1016/j.physbeh.2007.05.023.
8. Jacobs, J.V., and Horak, F.B. (2007). Cortical control of postural responses. J. Neural. Transm. *114*, 1339–1348. https://doi.org/10.1007/s00702-007-0657-0.
9. MacKinnon, C.D. (2018). Sensorimotor anatomy of gait, balance, and falls. Handb. Clin. Neurol. *159*, 3–26. https://doi.org/10.1016/B978-0-444-63916-5.00001-X.
10. Murray, A.J., Croce, K., Belton, T., Akay, T., and Jessell, T.M. (2018). Balance Control Mediated by Vestibular Circuits Directing Limb Extension or Antagonist Muscle Co-activation. Cell Rep. *22*, 1325–1338. https://doi.org/10.1016/j.celrep.2018.01.009.
11. Beloozerova, I.N., Sirota, M.G., Orlovsky, G.N., and Deliagina, T.G. (2005). Activity of pyramidal tract neurons in the cat during postural corrections. J. Neurophysiol. *93*, 1831–1844. https://doi.org/10.1152/jn.00577.2004.
12. Carpenter, M.G., Frank, J.S., Silcher, C.P., and Peysar, G.W. (2001). The influence of postural threat on the control of upright stance. Exp. Brain Res. *138*, 210–218. https://doi.org/10.1007/s002210100681.
13. Carpenter, M.G., Frank, J.S., and Silcher, C.P. (1999). Surface height effects on postural control: a hypothesis for a stiffness strategy for stance. J. Vestib. Res. *9*, 277–286.
14. Horslen, B.C., Zaback, M., Inglis, J.T., Blouin, J.S., and Carpenter, M.G. (2018). Increased human stretch reflex dynamic sensitivity with height-induced postural threat. J. Physiol. *596*, 5251–5265. https://doi.org/10.1113/JP276459.
15. Horslen, B.C., Murnaghan, C.D., Inglis, J.T., Chua, R., and Carpenter, M.G. (2013). Effects of postural threat on spinal stretch reflexes: evidence for increased muscle spindle sensitivity? J. Neurophysiol. *110*, 899–906. https://doi.org/10.1152/jn.00065.2013.
16. Horslen, B.C., Dakin, C.J., Inglis, J.T., Blouin, J.S., and Carpenter, M.G. (2014). Modulation of human vestibular reflexes with increased postural threat. J. Physiol. *592*, 3671–3685. https://doi.org/10.1113/jphysiol.2014.270744.
17. Naranjo, E.N., Cleworth, T.W., Allum, J.H.J., Inglis, J.T., Lea, J., Westerberg, B.D., and Carpenter, M.G. (2016). Vestibulo-spinal and vestibulo-ocular reflexes are modulated when standing with increased postural threat. J. Neurophysiol. *115*, 833–842. https://doi.org/10.1152/jn.00626.2015.
18. Naranjo, E.N., Allum, J.H.J., Inglis, J.T., and Carpenter, M.G. (2015). Increased gain of vestibulospinal potentials evoked in neck and leg muscles when standing under height-induced postural threat. Neuroscience *293*, 45–54. https://doi.org/10.1016/j.neuroscience.2015.02.026.
19. Horak, F.B., Nutt, J.G., and Nashner, L.M. (1992). Postural inflexibility in parkinsonian subjects. J. Neurol. Sci. *111*, 46–58. https://doi.org/10.1016/0022-510x(92)90111-w.
20. Rinalduzzi, S., Trompetto, C., Marinelli, L., Alibardi, A., Missori, P., Fattapposta, F., Pierelli, F., and Currà, A. (2015). Balance dysfunction in Parkinson's disease. BioMed Res. Int. *2015*, 434683, https://doi.org/10.1155/2015/434683.
21. Ennaceur, A. (2011). Omission of the habituation procedure in the acquisition of a working memory task - evidence from Balb/c, C57/BL6J, and CD-1 mice. Behav. Brain Res. *223*, 203–210. https://doi.org/10.1016/j.bbr.2011.04.035.
22. Crawley, J., and Goodwin, F.K. (1980). Preliminary report of a simple animal behavior model for the anxiolytic effects of benzodiazepines. Pharmacol. Biochem. Behav. *13*, 167–170. https://doi.org/10.1016/0091-3057(80)90067-2.
23. Graeff, F.G., Netto, C.F., and Zangrossi, H., Jr. (1998). The elevated T-maze as an experimental model of anxiety. Neurosci. Biobehav. Rev. *23*, 237–246. https://doi.org/10.1016/s0149-7634(98)00024-4.
24. Kulesskaya, N., and Voikar, V. (2014). Assessment of mouse anxiety-like behavior in the light-dark box and open-field arena: role of equipment and procedure. Physiol. Behav. *133*, 30–38. https://doi.org/10.1016/j.physbeh.2014.05.006.
25. Adhikari, A. (2014). Distributed circuits underlying anxiety. Front. Behav. Neurosci. *8*, 112. https://doi.org/10.3389/fnbeh.2014.00112.
26. Witts, E.C., and Murray, A.J. (2019). Vestibulospinal contributions to mammalian locomotion. Curr. Opin. Physiol. *8*, 56–62.
27. Chow, B.Y., Han, X., Dobry, A.S., Qian, X., Chuong, A.S., Li, M., Henninger, M.A., Belfort, G.M., Lin, Y., Monahan, P.E., and Boyden, E.S. (2010). High-performance genetically targetable optical neural silencing by light-driven proton pumps. Nature *463*, 98–102. https://doi.org/10.1038/nature08652.

CellPress
OPEN ACCESS

28. Pompeiano, O. (2001). Role of the locus coeruleus in the static and dynamic control of posture. Arch. Ital. Biol. *139*, 109–124.

29. Pompeiano, O. (1989). Relationship of noradrenergic locus coeruleus neurones to vestibulospinal reflexes. Prog. Brain Res. *80*, 329–343. https://doi.org/10.1016/s0079-6123(08)62228-1.

30. Fung, S.J., Manzoni, D., Chan, J.Y., Pompeiano, O., and Barnes, C.D. (1991). Locus coeruleus control of spinal motor output. Prog. Brain Res. *88*, 395–409. https://doi.org/10.1016/s0079-6123(08)63825-x.

31. Carter, M.E., Yizhar, O., Chikahisa, S., Nguyen, H., Adamantidis, A., Nishino, S., Deisseroth, K., and de Lecea, L. (2010). Tuning arousal with optogenetic modulation of locus coeruleus neurons. Nat. Neurosci. *13*, 1526–1533. https://doi.org/10.1038/nn.2682.

32. Sara, S.J. (2009). The locus coeruleus and noradrenergic modulation of cognition. Nat. Rev. Neurosci. *10*, 211–223. https://doi.org/10.1038/nrn2573.

33. Miles, G.B., and Sillar, K.T. (2011). Neuromodulation of vertebrate locomotor control networks. Physiology *26*, 393–411. https://doi.org/10.1152/physiol.00013.2011.

34. Hultborn, H., and Kiehn, O. (1992). Neuromodulation of vertebrate motor neuron membrane properties. Curr. Opin. Neurobiol. *2*, 770–775. https://doi.org/10.1016/0959-4388(92)90132-5.

35. Jaim-Etcheverry, G., and Zieher, L.M. (1980). DSP-4: a novel compound with neurotoxic effects on noradrenergic neurons of adult and developing rats. Brain Res. *188*, 513–523. https://doi.org/10.1016/0006-8993(80)90049-9.

36. Choudhary, P., Pacholko, A.G., Palaschuk, J., and Bekar, L.K. (2018). The locus coeruleus neurotoxin, DSP4, and/or a high sugar diet induce behavioral and biochemical alterations in wild-type mice consistent with Alzheimers related pathology. Metab. Brain Dis. *33*, 1563–1571. https://doi.org/10.1007/s11011-018-0263-x.

37. Ross, S.B., and Stenfors, C. (2015). DSP4, a selective neurotoxin for the locus coeruleus noradrenergic system. A review of its mode of action. Neurotox. Res. *27*, 15–30. https://doi.org/10.1007/s12640-014-9482-z.

38. Jones, M.D., and Hess, E.J. (2003). Norepinephrine regulates locomotor hyperactivity in the mouse mutant coloboma. Pharmacol. Biochem. Behav. *75*, 209–216. https://doi.org/10.1016/s0091-3057(03)00073-x.

39. Brunello, N., Blier, P., Judd, L.L., Mendlewicz, J., Nelson, C.J., Souery, D., Zohar, J., and Racagni, G. (2003). Noradrenaline in mood and anxiety disorders: basic and clinical studies. Int. Clin. Psychopharmacol. *18*, 191–202. https://doi.org/10.1097/00004850-200307000-00001.

40. Schuerger, R.J., and Balaban, C.D. (1993). Immunohistochemical demonstration of regionally selective projections from locus coeruleus to the vestibular nuclei in rats. Exp. Brain Res. *92*, 351–359. https://doi.org/10.1007/BF00229022.

41. Lyons, W.E., Fritschy, J.M., and Grzanna, R. (1989). The noradrenergic neurotoxin DSP-4 eliminates the coeruleospinal projection but spares projections of the A5 and A7 groups to the ventral horn of the rat spinal cord. J. Neurosci. *9*, 1481–1489.

42. Hwang, D.Y., Carlezon, W.A., Jr., Isacson, O., and Kim, K.S. (2001). A high-efficiency synthetic promoter that drives transgene expression selectively in noradrenergic neurons. Hum. Gene Ther. *12*, 1731–1740. https://doi.org/10.1089/104303401750476230.

43. Murray, A.J., Sauer, J.F., Riedel, G., McClure, C., Ansel, L., Cheyne, L., Bartos, M., Wisden, W., and Wulff, P. (2011). Parvalbumin-positive CA1 interneurons are required for spatial working but not for reference memory. Nat. Neurosci. *14*, 297–299. https://doi.org/10.1038/nn.2751.

44. Bolton, D.A.E. (2015). The role of the cerebral cortex in postural responses to externally induced perturbations. Neurosci. Biobehav. Rev. *57*, 142–155. https://doi.org/10.1016/j.neubiorev.2015.08.014.

45. McCollum, G., Shupert, C.L., and Nashner, L.M. (1996). Organizing sensory information for postural control in altered sensory environments. J. Theor. Biol. *180*, 257–270. https://doi.org/10.1006/jtbi.1996.0101.

46. Balaban, C.D. (2002). Neural substrates linking balance control and anxiety. Physiol. Behav. *77*, 469–475. https://doi.org/10.1016/s0031-9384(02)00935-6.

47. Ross, J.A., and Van Bockstaele, E.J. (2020). The Locus Coeruleus-Norepinephrine System in Stress and Arousal: Unraveling Historical, Current, and Future Perspectives. Front. Psychiatry *11*, 601519, https://doi.org/10.3389/fpsyt.2020.601519.

48. Pompeiano, O., Horn, E., and d'Ascanio, P. (1991). Locus coeruleus and dorsal pontine reticular influences on the gain of vestibulospinal reflexes. Prog. Brain Res. *88*, 435–462. https://doi.org/10.1016/s0079-6123(08)63827-3.

49. Wu, M.F., Gulyani, S.A., Yau, E., Mignot, E., Phan, B., and Siegel, J.M. (1999). Locus coeruleus neurons: cessation of activity during cataplexy. Neuroscience *91*, 1389–1399. https://doi.org/10.1016/s0306-4522(98)00600-9.

50. Kirsten, E.B., and Sharma, J.N. (1976). Characteristicas and response differences to iontophoretically applied norepinephrine, D-amphetamine and acetylcholine on neurons in the medial and lateral vestibular nuclei of the cat. Brain Res. *112*, 77–90. https://doi.org/10.1016/0006-8993(76)90335-8.

51. Suárez-Pereira, I., Llorca-Torralba, M., Bravo, L., Camarena-Delgado, C., Soriano-Mas, C., and Berrocoso, E. (2022). The Role of the Locus Coeruleus in Pain and Associated Stress-Related Disorders. Biol. Psychiatry *91*, 786–797. https://doi.org/10.1016/j.biopsych.2021.11.023.

52. Schwarz, L.A., Miyamichi, K., Gao, X.J., Beier, K.T., Weissbourd, B., DeLoach, K.E., Ren, J., Ibanes, S., Malenka, R.C., Kremer, E.J., and Luo, L. (2015). Viral-genetic tracing of the input-output organization of a central noradrenaline circuit. Nature *524*, 88–92. https://doi.org/10.1038/nature14600.

53. Kiehn, O., Sillar, K.T., Kjaerulff, O., and McDearmid, J.R. (1999). Effects of Noradrenaline on Locomotor Rhythm–Generating Networks in the Isolated Neonatal Rat Spinal. Cord Journal of Neurophysiology 82, 741–746.

54. McLean D.L., Sillar K.T.. (2003). Spinal and supraspinal functions of noradrenaline in the frog embryo: consequences for motor behaviour. J Physiol. 551. 2, 575-587. doi: 10.1113/jphysiol.2003.045229.

55. Harro, J., Meriküla, A., Lepiku, M., Modiri, A.R., Rinken, A., and Oreland, L. (2000). Lesioning of locus coeruleus projections by DSP-4 neurotoxin treatment: effect on amphetamine-induced hyperlocomotion and dopamine D2 receptor binding in rats. Pharmacol. Toxicol. *86*, 197–202. https://doi.org/10.1034/j.1600-0773.2000.d01-35.x.

56. Bent, L.R., Inglis, J.T., and McFadyen, B.J. (2004). When is vestibular information important during walking? J. Neurophysiol. *92*, 1269–1275. https://doi.org/10.1152/jn.01260.2003.

57. Bent, L.R., McFadyen, B.J., and Inglis, J.T. (2004). Is the use of vestibular information weighted differently across the initiation of walking? Exp. Brain Res. *157*, 407–416. https://doi.org/10.1007/s00221-004-1854-9.

58. Bent, L.R., McFadyen, B.J., and Inglis, J.T. (2005). Vestibular contributions during human locomotor tasks. Exerc. Sport Sci. Rev. *33*, 107–113.

59. Gesi, M., Soldani, P., Giorgi, F.S., Santinami, A., Bonaccorsi, I., and Fornai, F. (2000). The role of the locus coeruleus in the development of Parkinson's disease. Neurosci. Biobehav. Rev. *24*, 655–668. https://doi.org/10.1016/s0149-7634(00)00028-2.

60. Grimbergen, Y.A.M., Langston, J.W., Roos, R.A.C., and Bloem, B.R. (2009). Postural instability in Parkinson's disease: the adrenergic hypothesis and the locus coeruleus. Expert Rev. Neurother. *9*, 279–290. https://doi.org/10.1586/14737175.9.2.279.

61. Rinalduzzi, S., and Curra, A. (2015). "Postural strategies assessed with inertial sensors in healthy and Parkinson subjects" by C. Baston et al. Mancini, M., Schoneburg, B., Horak, F. and Rocchi, L.[Gait Posture 40 (2014) 70-75]: Really a new method to analyze postural strategy? Gait Posture *41*, 744. https://doi.org/10.1016/j.gaitpost.2014.12.020.

62. Chastan, N., Debono, B., Maltête, D., and Weber, J. (2008). Discordance between measured postural instability and absence of clinical symptoms in Parkinson's disease patients in the early stages of the disease. Mov. Disord. *23*, 366–372. https://doi.org/10.1002/mds.21840.

63. Park, H., Satoh, H., Miki, A., Urushihara, H., and Sawada, Y. (2015). Medications associated with falls in older people: systematic review of publications from a recent 5-year period. Eur. J. Clin. Pharmacol. *71*, 1429–1440. https://doi.org/10.1007/s00228-015-1955-3.

64. Devos, D., Bordet, R., and Defebvre, L. (2010). [Pharmacological hypotheses and therapeutic strategies for gait disorders in Parkinson's disease]. Rev. Neurol. *166*, 168–177. https://doi.org/10.1016/j.neurol.2009.07.017.

65. Devos, D., Defebvre, L., and Bordet, R. (2010). Dopaminergic and non-dopaminergic pharmacological hypotheses for gait disorders in Parkinson's disease. Fundam. Clin. Pharmacol. *24*, 407–421. https://doi.org/10.1111/j.1472-8206.2009.00798.x.

66. Yssel, J.D., O'Neill, E., Nolan, Y.M., Connor, T.J., and Harkin, A. (2018). Treatment with the noradrenaline re-uptake inhibitor atomoxetine alone and in combination with the alpha2-adrenoceptor antagonist idazoxan attenuates loss of dopamine and associated motor deficits in the LPS inflammatory rat model of Parkinson's disease. Brain Behav. Immun. *69*, 456–469. https://doi.org/10.1016/j.bbi.2018.01.004.

67. Valentino, R.J., and Van Bockstaele, E. (2008). Convergent regulation of locus coeruleus activity as an adaptive response to stress. Eur. J. Pharmacol. *583*, 194–203. https://doi.org/10.1016/j.ejphar.2007.11.062.

68. McCall, J.G., Al-Hasani, R., Siuda, E.R., Hong, D.Y., Norris, A.J., Ford, C.P., and Bruchas, M.R. (2015). CRH Engagement of the Locus Coeruleus Noradrenergic System Mediates Stress-Induced Anxiety. Neuron *87*, 605–620. https://doi.org/10.1016/j.neuron.2015.07.002.

69. Poe, G.R., Foote, S., Eschenko, O., Johansen, J.P., Bouret, S., Aston-Jones, G., Harley, C.W., Manahan-Vaughan, D., Weinshenker, D., Valentino, R., et al. (2020). Locus coeruleus: a new look at the blue spot. Nat. Rev. Neurosci. *21*, 644–659. https://doi.org/10.1038/s41583-020-0360-9.

70. Akay, T., Tourtellotte, W.G., Arber, S., and Jessell, T.M. (2014). Degradation of mouse locomotor pattern in the absence of proprioceptive sensory feedback. Proc. Natl. Acad. Sci. USA *111*, 16877–16882. https://doi.org/10.1073/pnas.1419045111.

71. Murray, A.J., Woloszynowska-Fraser, M.U., Ansel-Bollepalli, L., Cole, K.L.H., Foggetti, A., Crouch, B., Riedel, G., and Wulff, P. (2015). Parvalbumin-positive interneurons of the prefrontal cortex support working memory and cognitive flexibility. Sci. Rep. *5*, 16778, https://doi.org/10.1038/srep16778.

72. Han, X., Chow, B.Y., Zhou, H., Klapoetke, N.C., Chuong, A., Rajimehr, R., Yang, A., Baratta, M.V., Winkle, J., Desimone, R., and Boyden, E.S. (2011). A high-light sensitivity optical neural silencer: development and application to optogenetic control of non-human primate cortex. Front. Syst. Neurosci. *5*, 18. https://doi.org/10.3389/fnsys.2011.00018.

73. McClure, C., Cole, K.L.H., Wulff, P., Klugmann, M., and Murray, A.J. (2011). Production and titering of recombinant adeno-associated viral vectors. J. Vis. Exp. e3348. https://doi.org/10.3791/3348.

74. Han, Z., Luo, N., Kou, J., Li, L., Xu, Z., Wei, S., Wu, Y., Wang, J., Ye, C., Lin, K., and Xu, F. (2022). Brain-wide TVA compensation allows rabies virus to retrograde target cell-type-specific projection neurons. Mol. Brain *15*, 13. https://doi.org/10.1186/s13041-022-00898-8.

75. Zhu, X., Lin, K., Liu, Q., Yue, X., Mi, H., Huang, X., He, X., Wu, R., Zheng, D., Wei, D., et al. (2020). Rabies Virus Pseudotyped with CVS-N2C Glycoprotein as a Powerful Tool for Retrograde Neuronal Network Tracing. Neurosci. Bull. *36*, 202–216. https://doi.org/10.1007/s12264-019-00423-3.

76. Ganley, R.P., Werder, K., Wildner, H., and Zeilhofer, H.U. (2021). Spinally projecting noradrenergic neurons of the locus coeruleus display resistance to AAV2retro-mediated transduction. Mol. Pain *17*, 17448069211037887, https://doi.org/10.1177/17448069211037887.

STAR★METHODS

KEY RESOURCES TABLE

REAGENT or RESOURCE	SOURCE	IDENTIFIER
Antibodies		
Rabbit anti tyrosine hydroxylase	Abcam	AB112; RRID:AB_297840
Goat anti GFP	Abcam	AB6673; RRID:AB_305643
Bacterial and virus strains		
AAV PRSx8-GFP	Murray Lab	N/A
AAV PRSx8-Cre	Murray Lab	N/A
AAV CAGG-Flex-TeLC-GFP	Murray Lab	N/A
AAV CAGG-Flex-GFP	Murray Lab	N/A
Chemicals, peptides, and recombinant proteins		
DSP-4	Sigma	C8417
Deposited data		
Raw data	This paper	https://osf.io/3sqcn/

RESOURCE AVAILABILITY

Lead contact

Further information and requests for resources and reagents should be directed to and will be fulfilled by the lead contact, Dr. Andrew Murray (a.murray@ucl.ac.uk).

Materials availability

Plasmids generated in this study are available on request from the lead contact.

Data and code availability

Source files for data reported in this paper are available via Open Science Framework (https://osf.io/3sqcn/). This paper does not report original code. Any additional information required to reanalyze the data reported in this paper are available from the lead contact upon reasonable request.

EXPERIMENTAL MODEL AND STUDY PARTICIPANT DETAILS

All experiments were performed under UK Home Office license according to the United Kingdom Animals (Scientific Procedures) Act 1986. Both male and female C57BL/6J mice 12-20 weeks old were used. Different mice were used for each experiment (depicted by different figures in the manuscript) and N numbers throughout the manuscript refer to the number of mice unless otherwise stated. A total of 59 mice were used for the experiments in this manuscript.

METHOD DETAILS

Surgical procedures

EMG implantations

Bipolar EMG electrodes were fabricated and implanted into the hindlimb muscles gastrocnemius, tibialis anterior, semitendinosus and vastus laterals using techniques described previously.[70] Briefly, mice were anesthetized in isoflurane (4% induction; 0.5–2% maintenance). The neck and hindlimb were shaved with incisions made at the neck and directly above the muscles to be implanted. Custom made bipolar electrodes were passed under the skin from the neck to the hindlimb and implanted into the appropriate muscles. Animals were allowed to recover for at least three days before beginning behavioral experiments. Prior to beginning perturbation experiments animals walked on a treadmill at a constant speed of 0.3 m/s while EMG was recorded to ensure the EMG signals were of sufficient quality and the expected flexor-extensor alternation pattern was obtained.

Stereotaxic injections

Stereotaxic injections into the LVN were carried out as described previously.[10,71] Briefly, mice were anesthetized with isoflurane (4% induction; 1-2% maintenance). An incision was made in the skin above the scalp and bregma and lambda visualized. A small burr hole was made above the injection site and AAV was injected using a Nanoject II or Nanoject III (Drummond) and a pulled glass pipette. Stereotaxic coordinates relative to bregma were as follows: lateral vestibular nucleus, anterior/posterior -6.05 mm; lateral 1.38 mm; depth from brain surface -4.5, -4.4 and -4.3 mm with each depth receiving 100 nL of AAV. Locus coeruleus, anterior/posterior -5.4 mm; lateral 0.85 mm; depth -3.85, -3.75, 3.65 mm with each depth receiving 50 nL of AAV. Following AAV injection the skin was closed with Vicryl Rapide sutures. Behavioral experiments commenced no sooner than 14 days after AAV injection to allow sufficient time for the transgene to be expressed.

Behavioral procedures

Lateral perturbations

Mice were anaesthetized using isoflurane to attach recording connectors then placed in enclosures 10 cm by 6 cm with walls of either 3.5 cm or 7 cm in height. The enclosure was situated atop a treadmill such that it could move laterally as the treadmill belt rotated. A TTL pulse was used to induce a reproducible movement of the treadmill which caused a lateral displacement of the enclosure of 115 mm with a peak acceleration between 0.53 and 0.54 ms, reached 50 ms from movement onset, and the entire movement lasted 140 ms. The order of testing each mouse in the different wall heights was varied but the difference between the two groups was not tested statistically so an order effect cannot be completely ruled out. Mice were exposed to both left and right perturbations to prevent preparation of posture for a perturbation in a particular direction. For each trial, mice were exposed to 10 perturbations in each direction in a single session. Trials were monitored using an overhead camera (see recording techniques below) and only trials where the animal maintained a consistent starting position with the head and body clearly oriented to the front of the arena were analyzed. Partial obstruction of the overhead view of the animals by EMG wires and fiber optic cables prevented precise tracking of body displacement in the same animals that had EMG or fiber optic implants.

Treadmill locomotion

Locomotion was tracked on a custom-built treadmill (Electronics workshop, Zoological Institute, University of Cologne). Video recording of the right-side view of the mouse was carried out with a high speed camera (Ximea) recording at 200 frames per second. For incline running experiments the front of the treadmill apparatus was raised such that the treadmill belt had an upward gradient of 10°.

Open field

Mice were allowed to freely explore a square white Perspex arena for a period of 10 min while being recorded by an overhead video camera recording at 30 frames per second. To avoid effects of habituation on experiments mice were exposed to the arena for 10 min on the three days prior to experimental recordings.

Recording techniques

Muscle activity was amplified using custom built pre-amplifiers and amplifier (University of Cologne), digitized using a 1401-3 (Cambridge Electronic Design), and recorded using Spike2 (Cambridge Electronic Design). Video was captured using an overhead Logitech Brio Webcam and side mounted Doric Behavior Tracking camera. Reflective markers were attached to the enclosure and the mouse's head to track relative head position during perturbation. In some trials animals were also recorded in the arena for ~10 s before and after perturbations. The breathing rate of age and gender-matched animals was recorded for a period of 5 min to calculate breathing rate.

Viruses

For AAV constructs using the PRSx8 promoter, the PRSx8 promoter sequence based on Hwang et al.[42] was *de novo* synthesized (Life Technologies) and inserted into the pAM AAV genome vector[43] upstream from the transgene via KpnI and BamHI restriction sites. The sequence of the synthesized PRSx8 promoter was as follows:

5′-AGCTTCCGCTAGACAAATGTGATTACCCCCGCTAGACAAATGTGATTACCCGCGCTAGACAAATGTGATTACCCCGCTAGACAAATGTGATTACCCCCCGCTAGACAAATGTGATTACCCCCGCTAGACAAATGTGATTACCCGCGCTAGACAAATGTGATTACCCCGCTAGACAAATGTGATTACCCCCGACCAGGGCATAAATGGCCAGGTGGGACCAGAGAGCTCACCCCAGCCGACTCTAG-3’

Optogenetic inhibition was achieved with an AAV (DJ serotype) expressing archaerhodopsin (ArchT)[72] obtained via Addgene (plasmid #29778).

AAVs were packaged via calcium phosphate transfection of HEK293 cells as detailed in[73] and purified using an AAVpro purification kit (Takara Bio).

Retrograde labeling

We injected AAV2-Retro encoding cre under the control of the PRSx8 promoter, which, when injected into the LVN, should express cre in noradrenergic neurons projecting to the LC.

We concurrently injected a cre dependent GFP (AAV-DJ-Flex-GFP) directly into the locus coeruleus. Here, we would expect to label only those locus coeruleus neurons projecting to the LVN. However, despite repeated attempts we have been unable to label these neurons.

CellPress
OPEN ACCESS

In further control studies, we injected a AAV2-Retro-CAG-GFP into the LVN, which should label populations of neurons that innervate the LVN regardless of their neurotransmitter phenotype. Indeed, with this viral construct we did observe labeling throughout the brain but labeled cells in the LC were notably absent.

These results are in contrast to studies in our laboratory showing that injection of the non-viral retrograde tracer cholera toxin beta subunit (CTB) into the LVN results on labeling of LC neurons as well as published work from other groups using anterograde and retrograde tracers demonstrating a direct connection between these two regions.[40] Our hypothesis therefore is that AAV2-Retro is unable to transduce the terminals of locus coeruleus noradrenergic neurons. We have confirmed this hypothesis via the injection of AAV-Retro into the spinal cord and comparing the ratio of neurons transduced with AAV-Retro compared to (CTB) (Figure S4). CTB labeled similar number of neurons as AAV-Retro in the motor cortex, but 5- to 10-fold fewer neurons in the LVN, pontine reticular nucleus and locus coeruleus (n = 3 animals). Selective tropism of AAV2-Retro has been reported previously for multiple brain areas.[74,75] Other studies have also indicated locus coeruleus neurons are resistant to infection with AAV2-Retro.[76] The lack of tropism for these neurons is perhaps not surprising given the non-classical nature of noradrenergic synapses. In separate work we have been able to label LC neurons from the LC via the use of rabies virus, but the poor infection rate in these studies (2-3 per animal) precludes behavioral experiments.

Drugs

DSP-4 blocks noradrenaline transporters, which reduces levels of noradrenaline and eventually kills noradrenergic neurons. DSP-4 (Sigma C8417) was dissolved in sterile saline and injected intraperitoneally to produce a final concentration of 50 mg/kg. First injection was carried out immediately after recording control behavior with a second dose after 4 days. Behavioral testing was carried out 2-4 days after administration to ensure maximal decrease in noradrenergic neurons.[36]

Histology

After recording, mice were transcardialy perfused using ice-cold 4% paraformaldehyde (PFA). Brains were harvested and fixed in 4% PFA overnight before being transferred to phosphate buffer solution. 50 μm thick coronal brain sections were cut on a vibratome (Leica) and mounted on Superfrost+ glass slides for histological processing. Antibodies used were as follows: Rabbit to tyrosine hydroxylase (1:500) (Abcam, catalogue number AB112); Goat anti GFP (1:1000) (Abcam catalogue number ab16673). .

QUANTIFICATION AND STATISTICAL ANALYSIS

Data analysis

Video was analyzed using MaxTraq Software (Innovision Systems). For treadmill running the toe and ankle from the right hindlimb were tracked while the mouse matched the speed of the treadmill. To provide an internal measure of limb movement relative to the body the eye and tip of the ear were also tracked. Tracking data were exported to Microsoft Excel for plotting and further analysis. Conditions pre and post drugs were compared using student's paired T-test.

To measure head displacement during perturbation experiments video data were again analyzed using MaxTraq Software (Innovision Systems). A fixed point on the top of the animal's head was tracked along with a fixed point on the arena. To measure head displacement, the angle between the head and arena points was measured from the onset of the perturbation through to ~500 ms after the perturbation when the head had generally returned to its starting position.

EMG signals, along with a motor encoder from the moving platform, were recorded in Spike2, rectified and DC variation removed. Continuous recording of EMG signals throughout the whole experiment for each mouse showed clear responses to perturbation compared to a relatively stable baseline. Maximum amplitude responses within 100ms of perturbation were exported to Microsoft Excel. Student's t tests were used to compare the mean of these values between the two independent high and low wall conditions and for quantification of ablation efficiency. $p < 0.05$ was considered significant.

For analysis of respiration rate, breaths were counted by an experimenter naïve to the experimental condition.

Current Biology, Volume *33*

Supplemental Information

The locus coeruleus directs sensory-motor reflex amplitude across environmental contexts

Emily C. Witts, Miranda A. Mathews, and Andrew J. Murray

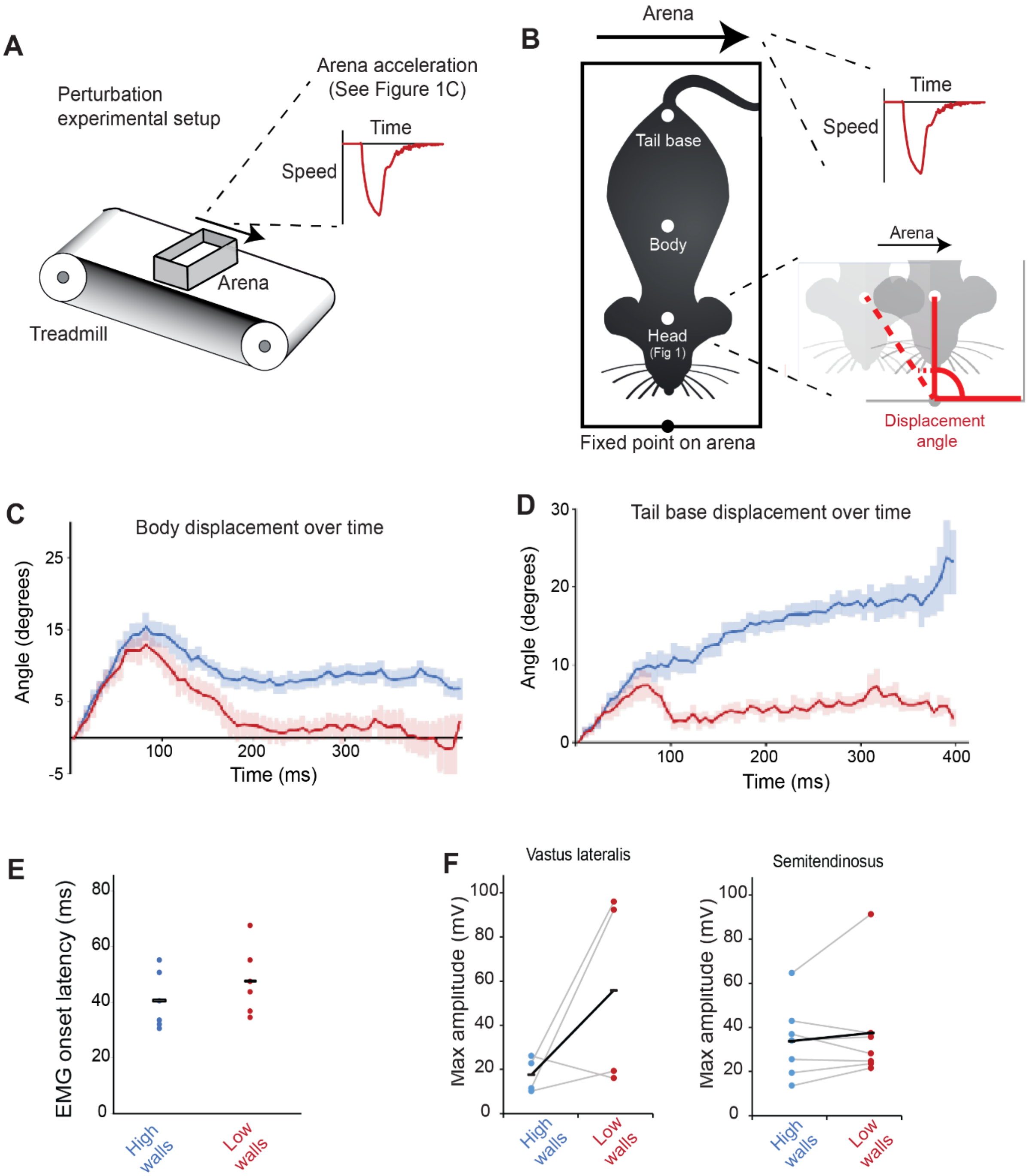

Figure S1: Behavioural setup to assess environmental effects on postural perturbations. Related to Figure 1. (A) Schematic showing experimental setup and perturbation acceleration. (B) Schematic showing placement of tracking dots and measurement of displacement angle. (C) Body displacement angle following perturbation. (D) Tail base displacement following perturbation. (E) Onset latency of muscle response following perturbation in high and low wall conditions. (F) Peak EMG amplitude of VL and ST muscles during perturbation in high and low walled conditions. Each point is the mean of trials from individual experimental animals with the overall mean represented by black lines.

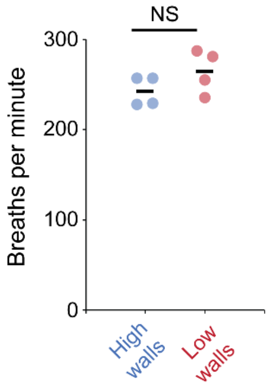

Figure S2: Respiratory rate of mice following lateral perturbations. Related to Figure 1B. Respiratory rate of animals after lateral perturbations.

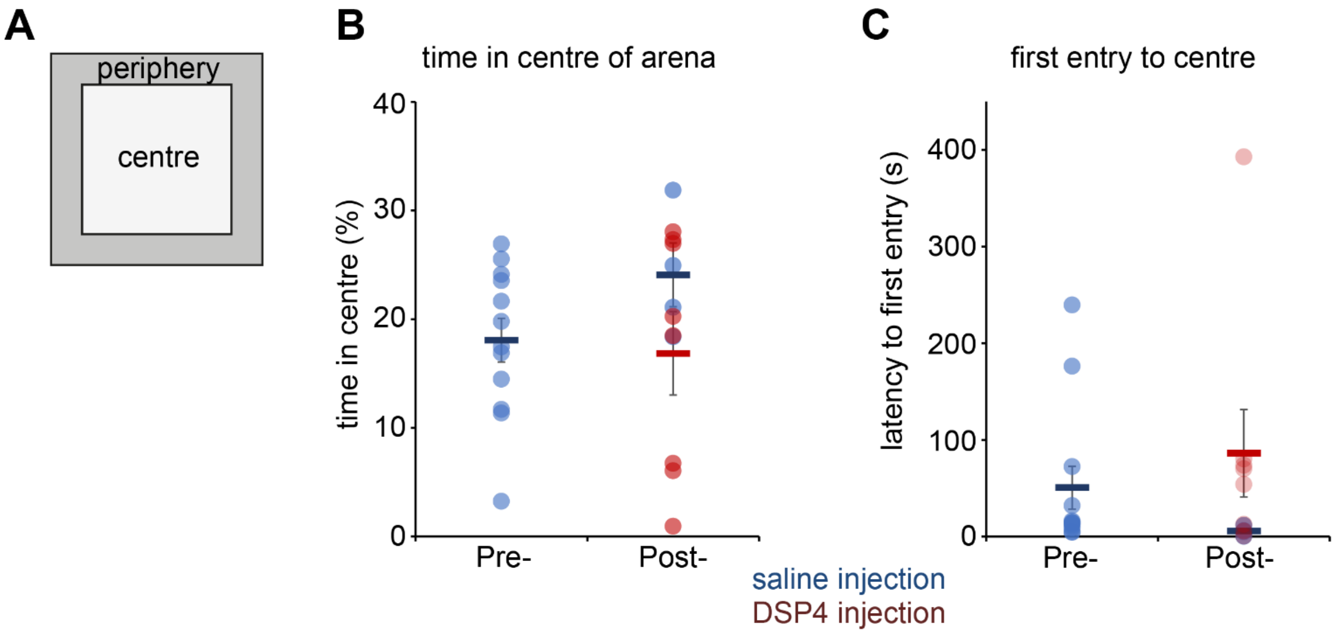

Figure S3: Open field locomotion in DSP4 and control animals. Related to Figure 3. (A) Schematic of open field arena. (B) Percentage of time spent in centre of arena pre- and post- DSP4 or saline injection. (C) Latency of first entry to centre of arena pre- and post- DSP4 or saline injection.

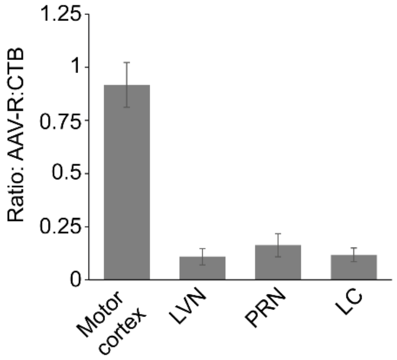

Figure S4: Tropism of AAV2-Retro for distinct populations of spinal projection neurons. Related to STAR methods. AAV-Retro and cholera toxin beta subunit (CTB) were injected into the lumbar spinal cord of adult mice. In motor cortex similar numbers of neurons were observed to contain both CTB and be transduced with AAV-Retro. However, in other regions, including the locus coeruleus, far fewer AAV-retro transduced neurons were observed indicating a lack of tropism for these neurons.
